# Evidence of chronic kidney disease in veterans with incident diabetes mellitus

**DOI:** 10.1371/journal.pone.0192712

**Published:** 2018-02-09

**Authors:** Justin Gatwood, Marie Chisholm-Burns, Robert Davis, Fridtjof Thomas, Praveen Potukuchi, Adriana Hung, Csaba P. Kovesdy

**Affiliations:** 1 University of Tennessee Health Science Center, College of Pharmacy, Memphis, TN, United States of America; 2 University of Tennessee Health Science Center, Center for Biomedical Informatics, Memphis, TN, United States of America; 3 University of Tennessee Health Science Center, Department of Preventive Medicine, Memphis, TN, United States of America; 4 Division of Nephrology, University of Tennessee Health Science Center, Memphis, TN, United States of America; 5 Vanderbilt University School of Medicine, Nashville, TN, United States of America; 6 VA Tennessee Valley Healthcare System, Nashville, TN, United States of America; 7 Memphis VA Medical Center, Memphis, TN, United States of America; Universita degli Studi di Perugia, ITALY

## Abstract

While chronic kidney disease (CKD) is regularly evaluated among patients with diabetes, kidney function may be significantly impaired before diabetes is diagnosed. Moreover, disparities in the severity of CKD in such a population are likely. This study evaluated the extent of CKD in a national cohort of 36,764 US veterans first diagnosed with diabetes between 2003 and 2013 and prior to initiating oral antidiabetic therapy. Evidence of CKD (any stage) at the time of diabetes diagnosis was determined using eGFR and urine-albumin-creatinine ratios, the odds of which were assessed using logistic regression controlling for patient characteristics. CKD was evident in 31.6% of veterans prior to being diagnosed with diabetes (age and gender standardized rates: 241.8 per 1,000 adults [overall] and 247.7 per 1,000 adult males), over half of whom had at least moderate kidney disease (stage 3 or higher). The odds of CKD tended to increase with age (OR: 1.88; 95% CI: 1.82–1.93), hemoglobin A1C (OR: 1.05; 95% CI: 1.04–1.06), systolic blood pressure (OR: 1.04; 95% CI: 1.027–1.043), and BMI (OR: 1.016; 95% CI: 1.011–1.020). Both Asian Americans (OR: 1.53; 95% CI: 1.15–2.04) and African Americans (OR: 1.11; 95% CI: 1.03–1.20) had higher adjusted odds of CKD compared to whites, and prevalence was highest in the Upper Midwest and parts of the Mid-South. Results suggest that evidence of CKD is common among veterans before a diabetes diagnosis, and certain populations throughout the country, such as minorities, may be afflicted at higher rates.

## Introduction

Approximately 10% of the general US population, more than 20 million people, has chronic kidney disease (CKD), and this condition is especially prevalent among patients with diabetes mellitus (DM) [1]. Specifically, an estimated one-third of adults with DM has CKD, and DM is the leading cause of CKD and end stage renal disease (ESRD) [1,2]. Several subgroups of the population, such as African Americans and Hispanics, experience higher rates or faster progression of CKD, and geographical variation in the prevalence of this condition has been suggested, although data on regional differences remain limited [3–6].

While multiple treatment options exist for patients with CKD, none are able to cure the patient of this affliction. However, multiple options do exist to delay progression of CKD, particularly for diabetic kidney disease, but early detection is paramount so that interventions may be introduced as soon as possible to effectively slow the disease’s progress [7–9]. While guidelines recommend screening for CKD in at-risk patients, in practice this is applied often only in patients with established DM [10,11]. Moreover, patients with DM often have one or more existing comorbid conditions but the extent to which these concomitant diseases may contribute to CKD is unclear. Better identification of patients who may have underlying or heightened risk for CKD may assist practitioners in recognizing and properly addressing kidney function among those who may not have otherwise been monitored.

The purpose of this investigation was to evaluate the extent to which CKD is present in patients prior to being diagnosed with and beginning treatment for DM. Using data from a nationwide electronic health record system, these analyses will add needed interpretation of the extent of racial and regional disparities among newly-diagnosed diabetes in whom evidence of CKD exists.

## Methods

### Study design and data source

This was a retrospective observational study using data from the VA Corporate Data Warehouse from 2002 through 2014. These data included extracts from the VA Decision Support System National Data Extracts, Inpatient and Outpatient Medical SAS Datasets (based on International Classification of Diseases, 9^th^ Revision, Clinical Modification [ICD-9-CM] codes), and the Vital Status Files. This study was reviewed and approved by institutional review boards at both the University of Tennessee Health Science Center and the Memphis VA Medical Center, and the need for consent was waived by these ethics committees.

The current analysis is based on a subset of a larger cohort that included incident cases of uncomplicated DM among veterans diagnosed between January 1, 2003 and December 31, 2012. In order to be eligible for the overall cohort, patients must have been 18 years and older, been diagnosed with diabetes (ICD-9-CD codes: 250.00 or 250.02) for the first time (i.e., no DM diagnostic codes in the year prior to the initial DM diagnosis), been prescribed an oral antidiabetic drug (OAD) as their first-line therapy for the first time (i.e., no OAD fills in the year prior to the first DM diagnosis code), and at least one year of data prior to and two years of data following their initial DM diagnosis. Patients were excluded if they were insulin dependent, had been diagnosed with a diabetes-related micro-vascular complication prior to or in conjunction with their DM diagnosis (ICD-9 codes listed in S1 Appendix), had been diagnosed with HIV at any point in their record, or had been diagnosed with malignant cancer prior to their initial DM diagnosis. This overall study cohort included 187,349 patients.

To address the objective of this research, the overall cohort was queried for requisite lab values recorded at visits to a VA facility. Criteria for inclusion at this stage of the analysis was the recording of two separate albuminuria and creatinine lab results prior to the diagnosis of DM at least 90 days apart; values closest to the index date (initial DM diagnosis) were used allowing for a window of 90 days past the index date for a lab value to be considered valid.

### Outcomes and covariates

The main outcome of this study was the presence of CKD in veterans prior to or at the time of their initial DM diagnosis. Evidence of CKD was determined in accordance with guidelines where normal kidney function was defined as a UACR ≤30 and an eGFR ≥60; CKD was categorized from stages 1 through, and also based on combinations of the levels of UACR (A1: <30 mg/g, A2: 30–300 mg/g and A3: >300 mg/g) and eGFR (G1: >90, G2: >60–90, G3: >30–60 and G4+G5: ≤30 ml/min/1.73m^2^) [12]. The odds of any stage of CKD (1–5) were modeled using patient characteristics within the VA records as predictors. The main covariates of interest were 1) race (White, African American, Asian American, Asian-Pacific Islander, and Native American) and 2) location of residence, which was defined as five geographic regions of the country (Northeast, Midwest, South, West, Other [US territories]). Additionally, other covariates included: age (at DM diagnosis and by 10-year bands), ethnicity (Hispanic or Non-Hispanic), hemoglobin A1C (as recorded closest to or at the DM diagnosis and by 1.0% increments), systolic blood pressure (at DM diagnosis and by 5.0 mmHg increments), body mass index (at DM diagnosis and by 1.0 kg/m^2^ increments), and the presence of several comorbid conditions/events included in the Deyo Charlson Comorbidity Index in the year prior to the initial DM diagnosis (cerebrovascular disease, congestive heart failure, peripheral artery disease, and myocardial infarction) [13]. Codes used to identify comorbid conditions/events are listed in the S1 Appendix.

### Statistical analyses

Data were described as means (standard deviations), medians (25^th^-75^th^ percentile), or counts (percent), as appropriate. Comparisons between categories of CKD stage were made using one-way ANOVA, correlation analysis, and the Cochran-Armitage test for trend. The adjusted odds of any stage of CKD (normal versus stages 1–5) were determined using multivariable logistic regression. To provide population-level estimates, crude prevalence rates were adjusted using population weights from the 2010 US census, and state-level estimates by race were derived using the 2011–2015 American Community Survey 5-Year Estimates [14,15]. A forward, step-wise model technique was used to determine the final set of predictors available or constructed within the patient records. Model fit was determined using Akaike’s information criterion and the Hosmer-Lemeshow Goodness-of-Fit Test. A sensitivity analysis defining CKD as stages 3a-5 was also conducted using logistic regression. All statistical tests used a two-tailed α = 0.05 level of significance and SAS Enterprise Guide version 7.1 (SAS, Cary, NC) was used for all statistical analyses.

To examine regional variation in CKD prevalence, patient data were categorized by VA markets, which are subdivisions within each of the 18 Veterans Integrated Service Networks (VISN) throughout the country. VA markets represent geographic areas sufficiently large to support a full healthcare delivery system, and this level of analysis allows for assessing patients whose care is more likely to be similar than at either a state or VISN level. Since the VA markets in general follow geographic boundaries of the US, this distinction also allows the broader assessment of CKD prevalence along traditional geographic distribution. ArcGIS version 10.4 (Esri, Redlands, CA) was used to generate the map and a projection using VA markets from fiscal year 2011 created the boundaries to more accurately reflect the underlying patient data used [16].

## Results

Out of the original cohort of 187,349 veterans, 36,764 remained eligible after considering the requisite lab values needed for this analysis. Across all patients included, mean eGFR was 78.5 mL/min/1.73 m^2^ (SD: 18.46) and median UACR was 8.1 mL/min/1.73 m^2^ (IQR: 4.1–20.7), and a higher proportion of patients with CKD were on either an angiotensin converting enzyme (ACE) inhibitor or an angiotensin II receptor blocker (ARB) by the time they were diagnosed with diabetes (p<0.0001). However, most patients with CKD were not on either therapy, irrespective of stage, and the proportion of patients on an ACE or ARB was markedly different based on year of diagnosis. Beginning in 2005, at least 31% of all patients (regardless of whether they had evidence of CKD) were on either therapy, but the proportion of those on an ACE or ARB remained consistently higher among patients with any stage of CKD, year-over-year. Table 1 describes the full characteristics of the sample.

**Table 1:** Baseline characteristics of incident diabetic patients overall and by CKD stage.

Characteristic	Overall (N = 36,764)	No CKD (N = 25,521)	Stage 1 (N = 1,837)	Stage 2 (N = 3,362)	Stage 3a (N = 4,663)	Stage 3b (N = 1,215)	Stage 4/5 (N = 166)	p-value
Age* *mean (SD)*	61.5 (10.22)	59.7 (9.53)	55.3 (8.55)	63.6 (9.74)	68.9 (8.86)	72.5 (9.12)	69.9 (11.11)	<0.0001
<34	276 (0.7)	230 (0.9)	35 (1.9)	10 (0.3)	0 (0)	1 (0.1)	0 (0)	
35–44	1,821 (4.9)	1,486 (5.8)	221 (12.0)	85 (2.5)	21 (0.5)	4 (0.3)	1 (0.6)	
45–54	6,527 (17.9)	5,291 (20.7)	509 (27.7)	515 (15.3)	205 (4.4)	38 (3.1)	14 (8.4)	
55–64	15,903 (43.3)	11,916 (46.7)	918 (50.0)	1,322 (39.3)	1,483 (31.8)	215 (17.7)	49 (29.5)	
65–74	8,320 (22.6)	5,090 (19.9)	143 (7.8)	965 (28.7)	1,675 (35.9)	410 (33.7)	37 (22.3)	
74–84	3,555 (9.7)	1,411 (5.5)	11 (0.6)	435 (12.9)	1,182 (25.4)	463 (38.1)	53 (31.9)	
85+	317 (0.9)	94 (0.4)	0 (0)	30 (0.9)	97 (2.1)	84 (6.9)	12 (7.2)	
Male	35,287 (95.9)	24,393 (95.6)	1,731 (94.2)	3,274 (97.4)	4,547 (97.5)	1,182 (97.3)	160 (96.4)	<0.0001
Race/Ethnicity								<0.0001
White	26,001 (70.7)	18,034 (70.7)	1,173 (63.9)	2,350 (69.9)	3,440 (73.8)	886 (72.9)	118 (71.1)	
American Indian	285 (0.8)	205 (0.8)	23 (1.3)	27 (0.8)	19 (0.4)	10 (0.8)	1 (0.6)	
Asian	262 (0.7)	184 (0.7)	17 (0.9)	26 (0.8)	30 (0.6)	4 (0.3)	1 (0.6)	
African American	4,681 (12.7)	3,481 (13.6)	353 (19.2)	420 (12.5)	347 (7.4)	70 (5.8)	10 (6.0)	
HPI	344 (0.9)	242 (0.9)	18 (1.0)	33 (1.0)	38 (0.8)	13 (1.1)	0 (0)	
Unknown	5,191 (14.1)	3,375 (13.2)	253 (13.8)	506 (15.1)	789 (16.9)	232 (19.1)	36 (21.7)	
Ethnicity								<0.0001
Hispanic	1,757 (4.8)	1,328 (5.2)	133 (7.2)	127 (3.8)	122 (2.6)	33 (2.7)	14 (8.4)	
Non-Hispanic	31,540 (85.8)	21,945 (86.0)	1,530 (83.3)	2,903 (86.3)	4,024 (86.3)	1,009 (83.0)	129 (77.7)	
Unknown	3,467 (9.4)	2,248 (8.8)	174 (9.5)	332 (9.9)	517 (11.1)	173 (14.2)	23 (13.9)	
Region								<0.0001
Northeast	6,040 (16.4)	4,247 (16.6)	252 (13.7)	518 (15.4)	805 (17.3)	198 (16.3)	20 (12.4)	
Midwest	10,113 (27.5)	6,778 (26.6)	430 (23.4)	985 (29.3)	1,468 (31.5)	396 (32.6)	56 (33.7)	
South	12,302 (33.5)	8,680 (34.0)	710 (38.6)	1,118 (33.3)	1,393 (29.9)	345 (28.4)	56 (33.7)	
West	8,144 (22.2)	5,712 (22.4)	434 (23.6)	719 (21.4)	977 (21.0)	271 (22.3)	31 (18.7)	
Other/Unknown	164 (0.4)	104 (0.4)	11 (0.6)	22 (0.7)	20 (0.4)	5 (0.4)	3 (1.8)	
Hemoglobin A1C* *mean (SD)*	7.4% (1.37)	7.3 (1.35)	7.9 (1.73)	7.5 (1.46)	7.2 (1.23)	7.1 (1.14)	7.2 (1.32)	<0.0001
BMI* *median (IQR)*	32.2 (28.9–36.3)	32.4 (29.0–36.4)	33.9 (30.1–38.4)	32.5 (29.1–36.6)	31.2 (28.2–34.8)	30.4 (27.5–34.0)	30.6 (27.7–34.7)	0.515
Underweight	220 (0.6)	148 (0.6)	10 (0.5)	25 (0.7)	31 (0.6)	6 (0.5)	0 (0)	
Normal	1,877 (5.1)	1,212 (4.8)	77 (4.2)	165 (4.9)	304 (6.5)	110 (9.1)	9 (5.4)	
Overweight	10,229 (27.8)	6,906 (27.1)	374 (20.4)	896 (26.7)	1,537 (33.0)	450 (37.0)	66 (39.8)	
Obese	24,438 (66.5)	17,255 (67.6)	1,376 (74.9)	2,276 (67.7)	2,791 (59.9)	649 (53.4)	91 (54.8)	
Systolic blood pressure	133.6 (16.29)	133.0 (15.55)	137.5 (17.43)	136.8 (17.99)	133.2 (17.33)	134.4 (18.67)	133.6 (18.31)	<0.0001
Charlson Comorbidity Index* *mean (SD)*	0.5 (0.65)	0.4 (0.63)	0.4 (0.61)	0.5 (0.68)	0.6 (0.73)	0.6 (0.71)	0.5 (0.69)	<0.0001
Comorbidities*								
CVD	2,236 (6.1)	1,349 (5.3)	77 (4.2)	243 (7.2)	426 (9.1)	125 (10.3)	2 (1.2)	<0.0001
MI	1,762 (4.8)	1,170 (4.6)	56 (3.0)	164 (4.9)	295 (6.3)	72 (5.9)	1 (0.6)	<0.0001
PAD	1,824 (5.0)	1,108 (4.3)	73 (4.0)	195 (5.8)	339 (7.3)	95 (7.8)	1 (0.6)	<0.0001
CHF	1,649 (4.5)	861 (3.4)	77 (4.2)	196 (5.8)	366 (7.8)	132 (10.8)	0 (0)	<0.0001
COPD	7,273 (19.8)	5,014 (19.7)	389 (21.2)	707 (21.0)	900 (19.3)	235 (19.3)	5 (3.0)	0.165
ACE/ARB†	11,072 (30.1)	7,160 (28.1)	474 (25.8)	1,122 (33.3)	1,790 (38.4)	473 (38.9)	53 (31.9)	
eGFR *mean (SD)*	78.5 (18.46)	84.1 (14.12)	100.6 (9.08)	74.8 (8.42)	53.9 (4.18)	39.6 (3.97)	20.8 (9.84)	<0.0001
UACR *median (IQR)*	8.1 (4.1–20.7)	6.2 (3.7–11.4)	65.0 (40.3–125.9)	62.6 (39.1–122.4)	10.0 (4.7–26.0)	18.0 (6.6–54.0)	29.8 (8.4–111.0)	<0.0001

Values provided are count (%) unless stated otherwise

Reported p-values references statistical differences (ANOVA or chi-square) between CKD stages

*By the time of DM diagnosis

† Single or combination therapy

HPI = Hawaiian/Pacific Islander; BMI = body mass index; CVD = cerebrovascular disease

MI = myocardial infraction; CHF = congestive heart failure; COPD = chronic obstructive pulmonary disease; PAD = peripheral artery disease; ACE = angiotensin-converting enzyme inhibitor; ARB = angiotensin II receptor blocker; eGFR = estimated glomerular filtration rate; UACR = urine albumin-creatinine ratio

**Fig 1:**
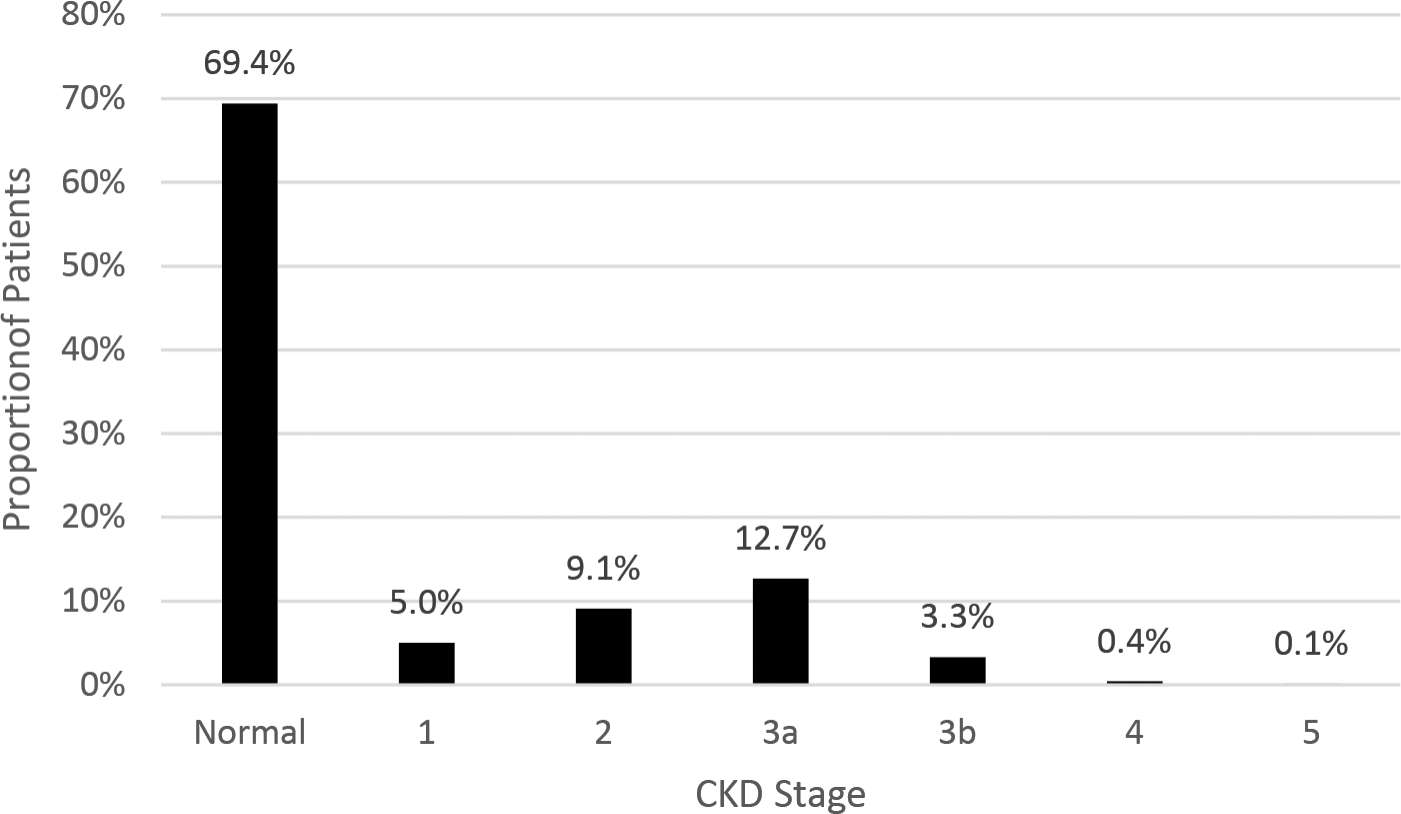
Prevalence of CKD prior to DM diagnosis.

Evidence of CKD (at any stage) was observed in 31.6% of veterans by the time they were diagnosed with DM, and over 16% exhibited at least moderately reduced eGFR (stages 3a-5) (Fig 1), which is over half of all the cases of CKD identified. Based on the levels of albuminuria and eGFR (Table 2), an approximately equal proportion of patients with CKD had normal eGFR level and mildly elevated UACR (stages A2G1 and A2G2: 13.2% of the entire cohort and 42.8% of those with CKD) and mildly decreased eGFR with no albuminuria (stage A1G3: 11.9% of the entire cohort and 38.9% of those with CKD). Relatively few patients had severe albuminuria (stage A3: 1.6% of the entire cohort and 5.2% of those with CKD) or moderately/severely decreased eGFR (stages G4+G5: 0.5% of the entire cohort and 1.4% of those with CKD).

**Table 2:** Study population laboratory values.

Full Study Population (N = 36,764)				
UACR (mg/g)	eGFR (ml/min/1.73m^2^)			
	>90	60–90	30–60	≤30
≤30	8,944 (24.3)	16,577 (45.1)	4,377 (11.9)	83 (0.2)
30 - ≤300	1,713 (4.7)	3,106 (8.5)	1,318 (3.6)	60 (0.2)
>300	124 (0.3)	256 (0.7)	183 (0.5)	23 (0.06)
CKD Patients (N = 11,243)				
UACR (mg/g)	eGFR (ml/min/1.73m^2^)			
	>90	60–90	30–60	≤30
≤30	0 (0)	0 (0)	4,377 (38.9)	83 (0.7)
30 - ≤300	1,713 (15.2)	3,106 (27.6)	1,318 (11.7)	60 (0.5)
>300	124 (1.1)	256 (2.3)	183 (1.6)	23 (0.2)

Values listed are counts (%)

Standardized to the general population by age and sex, the prevalence rate of any stage of CKD was approximately 241.8 cases per 1,000 people (95% CI: 218.3–265.4). Specific to men (96% of the sample), the age-adjusted rate was 247.7 per 1,000 people (95% CI: 230.2–265.2). Among the two largest racial categories, unadjusted values indicated that slightly more White veterans had evidence of CKD than African American veterans prior to being diagnosed with diabetes. Among patients with CKD, more advanced stage was moderately related to increased age (r = 0.48, p<0.0001) while those who self-identified as a racial minority (p<0.0001) were observed in higher proportions among less severe stages of CKD (Stages 1 or 2 versus 3 or higher). Additionally, hemoglobin A1C and systolic blood pressure tended to be lower among those with more advanced CKD (r = -0.18, p<0.0001; r = -0.09, p<0.0001) while the number of comorbidities tended to slightly increase with more severe CKD (r = 0.08, p<0.0001). No differences were observed between stages of CKD in terms of patient BMI.

**Fig 2:**
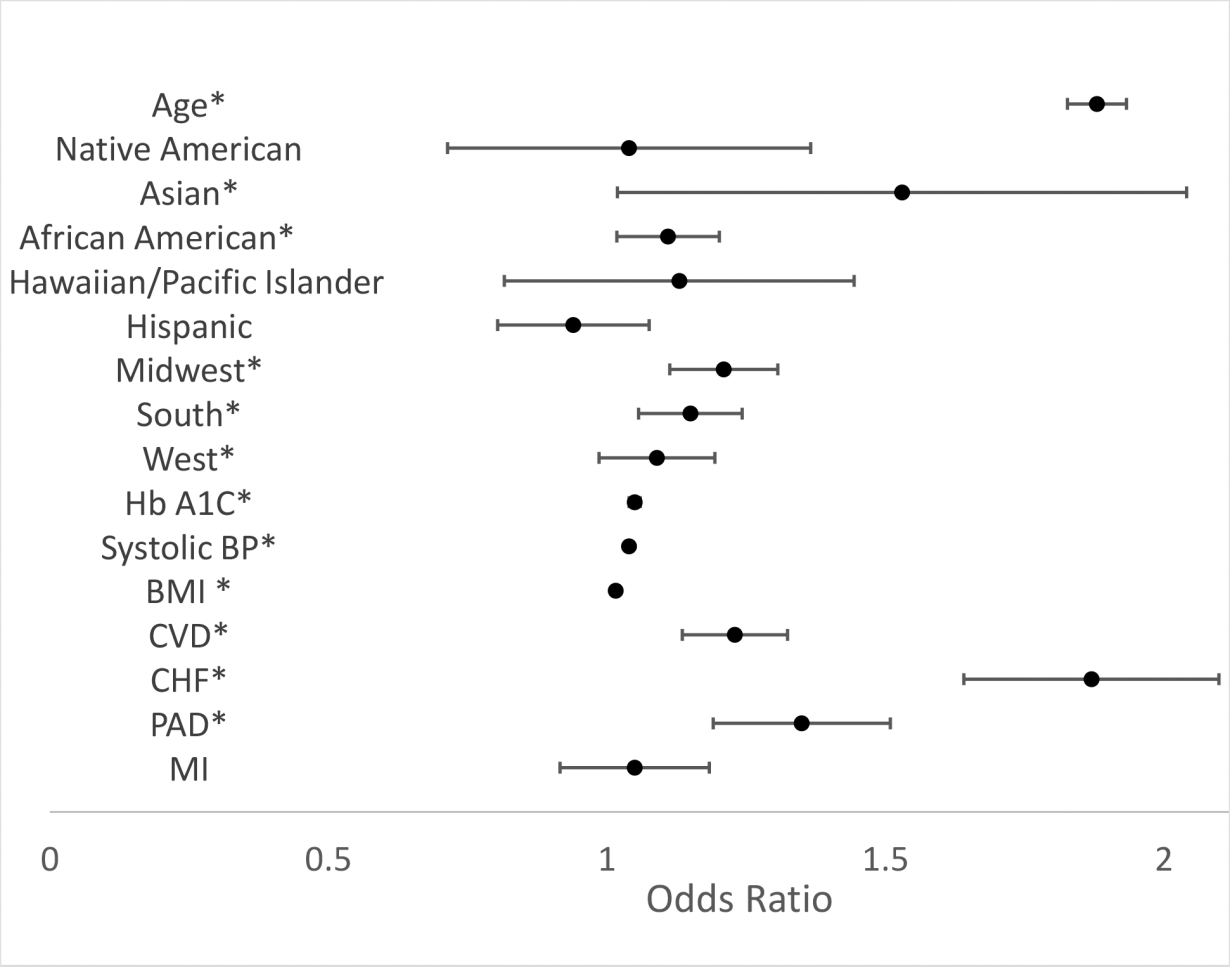
Odds of CKD prior to DM diagnosis. *p<0.05 Total sample in regression = 30,792, Age in 10-year bands, Hb A1C in 0.5% increments, blood pressure in 5.0 increments, and BMI in 1.0 increments, BMI = body mass index; Hb A1C = Hemoglobin A1C; CVD = cerebrovascular disease; CHF = congestive heart failure; PAD = peripheral artery disease; MI = myocardial infarction; API = Asian Pacific Islander, Reference categories: White (Race), Non-Hispanic (Ethnicity), Northeast (Region).

After adjusting for baseline characteristics, the odds of CKD (any stage) tended to increase with older age (OR: 1.88; 95% CI: 1.82–1.93), higher hemoglobin A1C (OR: 1.05; 95% CI: 1.04–1.06), higher systolic blood pressure (OR: 1.04; 95% CI: 1.027–1.043), and higher BMI (OR: 1.016; 95% CI: 1.011–1.020). The odds of CKD (Fig 2) were also higher among veterans with existing diagnoses for cerebrovascular disease (OR: 1.23; 95% CI: 1.11–1.36), congestive heart failure (OR: 1.87; 95% CI: 1.67–2.10), or peripheral artery disease (OR: 1.35; 95% CI: 1.21–1.51). Significant racial variation in the presence of CKD was also present. Compared to White veterans, both Asian Americans (OR: 1.53; 95% CI: 1.15–2.04) and African Americans (OR: 1.11; 95% CI: 1.03–1.20) had higher adjusted odds of any stage of CKD. Results of the sensitivity analysis were largely similar for all factors except race where the odds of CKD were no longer statistically higher for Asian or African America veterans (S2 Appendix).

**Fig 3:**
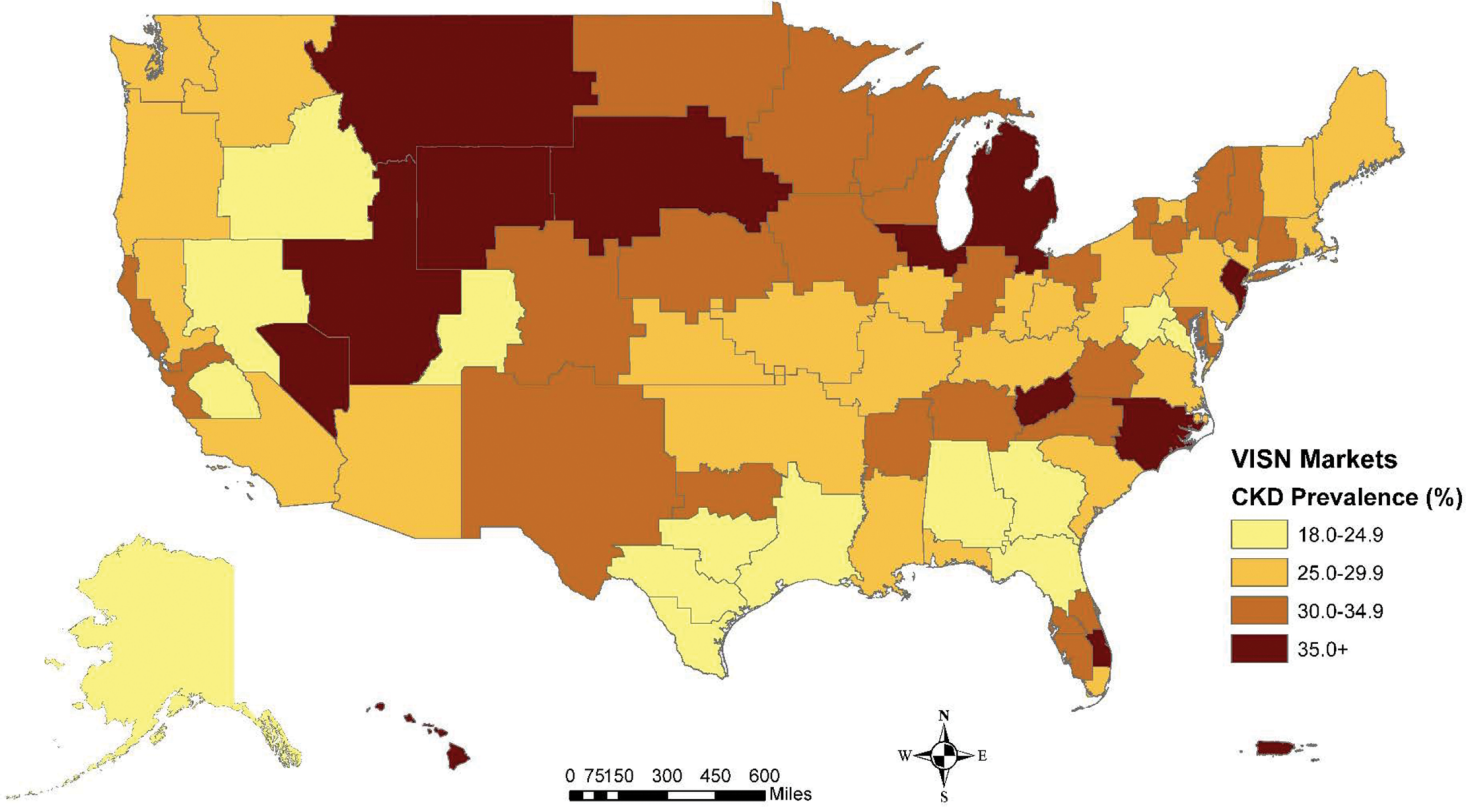
Geographic distribution of CKD in patients with incident diabetes.

Similarly, regional variation in the odds of CKD were noted. Using the northeastern portion of the country as a reference, all other regions of the United States had a higher odds of CKD: Midwest (OR: 1.21; 95% CI: 1.12–1.31), South (OR: 1.15; 95% CI: 1.07–1.24), and West (OR: 1.09; 95% CI: 1.01–1.19). Among the regions, the Midwest had the highest proportion of its veterans (32.9%) demonstrating CKD, and several pockets of the country exhibited markedly higher rates of CKD where unadjusted prevalence was at least 30% (Fig 3). Specifically, the Upper Midwest, Central and South Florida, and a band encompassing a portion of the Mid-South and North Carolina. Broken down by patient race, the highest prevalence of White veterans with evidence of CKD (32.7%) was observed in the Midwest while CKD prevalence was noticeably higher among Black veterans in the Midwest (27.6%) and South (26.6%). Specific to North Carolina, crude prevalence of CKD in White and African American veterans was 46.9% and 45.3%, respectively. Standardized by age, these equate to approximately 345.6 cases per 1,000 among Whites (95% CI: 239.8–451.4) and 452.5 cases per 1,000 among African Americans in the state (95% CI: 298.9–606.1).

## Discussion

Using data from the nationwide VA electronic health record system, we observed that among veterans who were eventually diagnosed with DM nearly one-third of them had evidence of CKD by the time of their initial DM diagnosis. Most of these patients had early stages of CKD (low grade albuminuria and/or mildly decreased eGFR). Similar to the general US population, significant racial disparities in the prevalence and severity of CKD were observed when adjusting for multiple patient characteristics. Additionally, some regional variation in CKD prevalence was noted, and several areas of the country had a particularly high number of cases.

According to data from the National Health and Nutrition Examination Survey (NHANES), the prevalence of CKD in patients with DM has been estimated to be 40% or higher while the rate among the non-diabetic population was estimated to be 11.3% [17,18, 19]. Although our estimate is slightly lower than this it is important to specify that these prior studies were conducted among patients with previously diagnosed DM- a known contributor to CKD, and a condition for which regular monitoring of kidney function is guideline-recommended [10]. The high prevalence of CKD in this incident diabetic cohort suggests that kidney damage could occur before diabetes is diagnosed; either because type 2 diabetes can be undiagnosed for a prolonged period of time and could cause organ damage even before a formal clinical diagnosis, or because of kidney damage caused by ancillary comorbid conditions that are common in this population. The association of CKD with known risk factors for kidney disease (such as older age, higher BMI, cardiovascular disease and African American race) suggests that among patients with type 2 DM CKD may develop as a result of various causes, and more specific diagnostic studies (e.g. kidney biopsy) would be needed to accurately differentiate diabetic and non-diabetic etiologies. Such an accurate determination is often not pursued in clinical practice absent etiology-specific therapeutic interventions, but it may become necessary if and when such therapies become available.

These results also highlight an opportunity for broadening screening efforts among patients that may be at increased risk for developing CKD, such as patients with high comorbidity burden. Moreover, it raises awareness to the importance of earlier identification of DM to prevent organ damage. Consequently, broader screening efforts, for both CKD and DM, should be advocated in order to limit kidney damage, particularly if cardiovascular comorbidities or other risk factors are known.

The observation that certain groups of patients, namely African American and Asian American veterans, had a higher adjusted odds of CKD reinforce findings from earlier studies demonstrating such a disparity in these populations, both in the general public and specific to veterans [2,20–23]. However, two key differences from previously published studies emerged. First, the distribution of disease severity (i.e., CKD stage) among African Americans in this study differed significantly from previous analyses of both veterans and the general US population. Specifically, our results suggested that the proportion of minorities declined as disease became more severe, which is contradictory to earlier evidence [21,24–26]. Secondly, research has suggested that Hispanics experience CKD at a rate higher than non-Hispanics in the US, but our analyses found no influence of this ethnicity on the odds of disease [6,21]. However, it is important to note that earlier studies have focused, in large part, on estimating differences in ESRD whereas our analysis assessed predictors of any CKD stage. Additionally, it is possible that these differences in findings are due, at least in part, to the point at which disease was identified. Our analyses focused on identifying CKD prior to a DM diagnosis- a condition known to advance kidney damage. While evidence suggests that African Americans and Hispanics experience a higher rate of progression of CKD compared to whites, it is possible that we isolated these patients at a point when disease had yet to significantly progress whereas other studies were less specific about the point at which CKD was identified in reference to a DM diagnosis, if one was even eventually given [6,27]. This may also explain the differences in odds ratios associated with patient race uncovered by the sensitivity analysis when the stage of disease determining CKD was elevated. Future studies may consider a longitudinal focus on patients of different races to more precisely identify whether temporality contributes to racial disparities in the progression to ESRD.

Our analysis also expanded the evidence behind geographic disparities in CKD by identifying sections of the country where prevalence was noticeably higher. Importantly, our study analyzed CKD prevalence using VA markets, which allowed us to investigate the presence of disease within regions of patients with more similar healthcare and characteristics-a more granular level than previous, similar assessments [2]. Moreover, we examined the prevalence of all stages of CKD to gain a better perspective on the entire course of disease rather than focusing solely on the occurrence of ESRD. The observation that rates of disease were particularly high in the Upper Midwest echo earlier findings where ESRD was particularly prevalent among African Americans in these states [2]. Additionally, the rates of CKD observed in North Carolina and parts of Florida reiterate the contention that residents of the southeastern United States are experiencing particular chronic conditions (e.g., diabetes and hypertension) at disparate rates [28,29]. However, these findings are in contrast to those reported by Plantinga and colleagues who observed a higher risk of ESRD among African American residents of the southeastern United States but no clear geographic pattern for other stages of CKD [30].

A variety of mechanisms have been hypothesized to contribute to disparities among patients with CKD with results consistently identifying lower socioeconomic status as a leading indicator of increased risk for disease and its progression to ESRD [30–34]. Additionally, some evidence has pointed to lack of insurance or access to care as being at least a partial contributor to the occurrence of CKD [35,36]; however, education was not independently associated with an increased risk for developing CKD [30]. While addressing socioeconomic factors poses a significant challenge, evidence behind the role of providing access, as well as the incorporation of population health management approaches, may indicate how disparities may be mitigated [31,37–39]. Systems like the VA, which ensure access to care for its patients, may be a good source of evidence in this country to examine whether the gap in CKD disparities can be narrowed simply by providing evidence-based care. While evidence suggests that significant CKD-related racial disparities exist in this population (which may be related to biological factors, including genetics), future studies involving veterans should incorporate deeper, and potentially longitudinal, analysis of socioeconomic factors that could be significantly hindering the efforts of care provided by the VA system [27,40].

This study was limited in several ways. First, only data from the VA’s electronic health record system were used to provide estimates of disease prevalence; therefore, results may not be generalizable to the general US or other populations. Secondly, data used were drawn straight from the electronic record without adjudication; therefore, errors made in the system while documenting laboratory values could have impacted our analyses. Also, exclusion criteria for the larger associated study that were not related to the current analysis may have introduced selection bias as two years’ worth of follow-up data were required for inclusion to the overall study cohort, meaning survival over this time period was required. Specific selection criteria that may limit interpretation includes the excluding of patients initiating diabetes treatment on insulin as well as those with existing diagnoses for microvascular complications, which removed patients with more advanced disease who were also potentially more likely to have some form of CKD. Therefore, our interpretation of the prevalence of CKD in the study population is likely biased by the fact that the analyzed cohort excluded sicker patients or those who died prior to meeting eligibility requirements. Additionally, while select cardiovascular events, such as myocardial infarction, were used as predictors of CKD in this analysis, a complex interrelationship exists between CKD and cardiovascular complications. Consequently, as these conditions existed simultaneously among the study population, we are not able to comment on the directionality of causation between CKD and the selected outcomes [41,42].

In spite of these limitations, the current study adds insight to the extent of both racial and regional variations in the prevalence of CKD while calling attention to the magnitude of this disease among patients prior to being diagnosed with DM. The current analysis suggests that expanded CKD and DM screening efforts may be necessary among veterans who are minorities, especially if cardiovascular comorbidities are present, in order to control the underlying kidney damage and narrow the disparities that exist in this population related to kidney disease, both independent of and in conjunction with DM.

## Supporting information

S1 Appendix Diagnostic and procedural codes. (DOCX)

S2 Appendix Logistic regression sensitivity analysis. (DOCX)

## References

[1] Centers for Disease Control and Prevention (CDC). National Chronic Kidney Disease Fact Sheet: General Information and National Estimates on Chronic Kidney Disease in the United States, 2014. Atlanta, GA: US Department of Health and Human Services, Centers for Disease Control and Prevention; 2014.

[2] Saran R, Li Y, Robinson B, Abbott KC, Agodoa LY, Ayanian J, Bragg-Gresham J, et al US Renal Data System 2015 Annual Data Report: epidemiology of kidney disease in the United States. Am J Kidney Dis. 2016; 67: S1–S434. 10.1053/j.ajkd.2015.12.014 PMC6643990 26925525

[3] Tanner RM, Gutierrez OM, Judd S, McClellan W, Bowling CB, Bradbury BD, et al Geographic variation in CKD prevalence and ESRD incidence in the United States: Results from the Reasons for Geographic and Racial Differences in Stroke (REGARDS) Study. Am J Kidney Dis. 2013; 61(3): 395–403. doi: 10.1053/j.ajkd.2012.10.018 23228944 10.1053/j.ajkd.2012.10.018 PMC3659181

[4] Bryson CL, Ross HJ, Boyko EJ, Young BA. Racial and ethnic variations in albuminuria in the US Third National Health and Nutrition Examination Survey (NHANES III) population: associations with diabetes and level of CKD. 2006; 48: 720–726. doi: 10.1053/j.ajkd.2006.07.023 17059991 10.1053/j.ajkd.2006.07.023

[5] Jones CA, Francis ME, Eberhardt MS, Chavers B, Coresh J, Engelgau M, et al Microalbuminuria in the US population: third National Health and Nutrition Examination Survey. Am J Kidney Dis. 2002; 39: 445–459. doi: 10.1053/ajkd.2002.31388 11877563 10.1053/ajkd.2002.31388

[6] Peralta CA, Shlipak MG, Fan D, Dongjie F, Ordonez J, Lash JP, Chertow GM, et al Risks for end-stage renal disease, cardiovascular events, and death in Hispanic versus non-Hispanic white adults with chronic kidney disease. J Am Soc Nephrol. 2006; 17: 2892–2899. doi: 10.1681/ASN.2005101122 16959827 10.1681/ASN.2005101122

[7] Lewis EJ, Hunsicker LG, Bain RP, Rohde RD for the Collaborative Study Group. The effect of angiotensin-converting-enzyme inhibition on diabetic nephropathy. N Engl J Med. 1993; 329: 1456–1462. doi: 10.1056/NEJM199311113292004 8413456 10.1056/NEJM199311113292004

[8] Brenner BM, Cooper ME, de Zeeuw D, Keane WF, Mitch WE, Parving HH, et al for the RENAAL Study Investigators. Effects of losartan on renal and cardiovascular outcomes in patients with type 2 diabetes and nephropathy. N Engl J Med. 2001; 345: 861–869. doi: 10.1056/NEJMoa011161 11565518 10.1056/NEJMoa011161

[9] Lewis EJ, Hunsicker LW, Clarke WR, Berl T, Pohl MA, Lewis JB, et al for the Collaborative Study Group. Renoprotective effect of the angiotensin-receptor antagonist irbesartan in patients with nephropathy due to type 2 diabetes. N Engl J Med. 2001; 345: 851–860. doi: 10.1056/NEJMoa011303 11565517 10.1056/NEJMoa011303

[10] KDOQI Clinical Practice Guidelines and Clinical Practice Recommendations for Diabetes and Chronic Kidney Disease. Am J Kidney Dis. 2007; 49(2): S12–S154. 17276798 10.1053/j.ajkd.2006.12.005

[11] Qaseem A, Hopkins RH, Sweet DE, Starkey M, Shekeel P for the Clinical Guidelines Committee of the American College of Physicians. Screening, monitoring, and treatment of stage 1 to 3 chronic kidney disease: A clinical practice guideline from the American College of Physicians. Ann Intern Med. 2013; 159(12): 835–847. doi: 10.7326/0003-4819-159-12-201312170-00726 24145991 10.7326/0003-4819-159-12-201312170-00726

[12] KDIGO 2012 clinical practice guideline for the evaluation and management of chronic kidney disease. Kidney Int Suppl. 2013; 3:1–150. 10.1038/ki.2013.243 23989362

[13] Deyo RA, Cherkin DC, Ciol MA. Adapting a clinical comorbidity index for use with ICD-9-CM administrative databases. J Clin Epidemiol. 1992; 45:613–619. 1607900 10.1016/0895-4356(92)90133-8

[14] United States Census Bureau. American FactFinder 2010 Census [Internet]. Washington, D.C.: U.S. Census Bureau; 2010 [cited December 27, 2016]. Available at: http://factfinder2.census.gov.

[15] United States Census Bureau. 2011–2015 American Community Survey 5-Year Estimates [Internet]. Washington, D.C.: U.S. Census Bureau; 2015 [cited January 4, 2017]. Available at: http://factfinder2.census.gov.

[16] USVA VHA Healthcare Services Geographies. FY2011 Q4 Markets, Geodatabase Feature Class. Washington, D.C.: Veterans Health Administration; 2013.

[17] Koro CE, Lee BH, Bowlin SJ. Antidiabetic medication use and prevalence of chronic kidney disease among patients with type 2 diabetes mellitus in the United States. Clin Ther. 2009; 31(11): 2608–2617. doi: 10.1016/j.clinthera.2009.10.020 20110005 10.1016/j.clinthera.2009.10.020

[18] Bailey RA, Wang Y, Zhu V, Rupnow MFT. Chronic kidney disease in US adults with type 2 diabetes: an updated national estimate of prevalence based on Kidney Disease: Improving Global Outcomes (KDIGO) staging. BMC Res Notes. 2014; 7: 415 doi: 10.1186/1756-0500-7-415 24990184 10.1186/1756-0500-7-415 PMC4091951

[19] Murphy D, McCulloch CE, Lin F, Banerjee T, Bragg-Gresham JL, Eberhardt MS, et al for the Centers for Disease Control and Prevention Chronic Kidney Disease Surveillance Team. Trends in prevalence of chronic kidney disease in the United States. Ann Intern Med. 2016; 165(7): 473–481. doi: 10.7326/M16-0273 27479614 10.7326/M16-0273 PMC5552458

[20] Peralta CA, Lin F, Shlipak MG, Siscovik D, Lewis C, Jacobs DR Jr, et al Race differences in prevalence of chronic kidney disease among young adults using creatinine-based glomerular filtration rate-estimating equations. Nephrol Dial Transplant. 2010; 25: 3934–3939. doi: 10.1093/ndt/gfq299 20519233 10.1093/ndt/gfq299 PMC3108366

[21] Derose SF, Rutkowski MP, Crooks PW, Shi JM, Wang JQ, Zalantar-Zadeh K, et al Racial differences in estimated GFR decline, ESRD, and mortality in an integrated health system. Am J Kidney Dis. 2013; 62: 236–244. doi: 10.1053/j.ajkd.2013.01.019 23499049 10.1053/j.ajkd.2013.01.019 PMC3723721

[22] Young BA, Maynard C, Boyko EJ. Racial differences in diabetic nephropathy, cardiovascular disease, and mortality in a national population of veterans. Diabetes Care. 2003; 26: 2392–2399. 12882868 10.2337/diacare.26.8.2392

[23] Kovesdy CP, Anderson JE, Derose SF, Kalantar-Zadeh K. Outcomes associated with race in males with nondialysis-dependent chronic kidney disease. Clin J Am Soc Nephrol. 2009; 4(5): 973–978. doi: 10.2215/CJN.06031108 19369403 10.2215/CJN.06031108 PMC2676182

[24] Nicholas SB, Kalantar-Zadeh K, Norris KC. Racial disparities in kidney disease outcomes. Seminars in nephrology. 2013; 33(5):409–415. doi: 10.1016/j.semnephrol.2013.07.002 24119846 10.1016/j.semnephrol.2013.07.002 PMC3983362

[25] Hsu CY, Lin F, Vittinghoff E, Shlipak MG. Racial differences in the progression from chronic renal insufficiency to end-stage renal disease in the United States. J Am Soc Nephrol. 2003; 14(11):2902–2907. 14569100 10.1097/01.asn.0000091586.46532.b4

[26] McClellan W, Warnock DG, McCLure L, Campbell RC, Newsome BB, Howard V, et al Racial differences in the prevalence of chronic kidney disease among participants in the Reasons for Geographic and Racial Differences in Stroke (REGARDS) cohort study. J Am Soc Nephrol. 2006; 17: 1710–1715. doi: 10.1681/ASN.2005111200 16641151 10.1681/ASN.2005111200

[27] Parsa A, Kao WH, Xie D, Astor BC, Li M, Hsu CY, et al APOL1 risk variants, race, and progression of chronic kidney disease. N Engl J Med. 2013; 369: 2183–2196. doi: 10.1056/NEJMoa1310345 24206458 10.1056/NEJMoa1310345 PMC3969022

[28] Barker LE, Kirtland KA, Gregg EW, Geiss LS, Thompson TJ. Geographic distribution of diagnosed diabetes in the U.S.: a diabetes belt. Am J Prev Med. 2011; 40: 434–439. doi: 10.1016/j.amepre.2010.12.019 21406277 10.1016/j.amepre.2010.12.019

[29] Howard VJ, Woolson RF, Egan BM, Nicholas JS, Adams RJ, Howard G, et al Prevalence of hypertension by duration and age at exposure to the stroke belt. J Am Soc Hypertens. 2010; 4: 32–41. doi: 10.1016/j.jash.2010.02.001 20374949 10.1016/j.jash.2010.02.001 PMC2872151

[30] Plantinga L, Howard VJ, Judd S, Muntner P, Tanner R, Rizk D, et al Association of duration of residence in the southeastern United States with chronic kidney disease may differ by race: The REasons for Geographic and Racial Differences in Stroke (REGARDS) cohort study. Int J Health Geogr. 2013; 12:17 doi: 10.1186/1476-072X-12-17 23518004 10.1186/1476-072X-12-17 PMC3606831

[31] Vart P, Gansevoort RT, Coresh J, Reijneveld SA, Bultmann U. Socioeconomic Measures and CKD in the United States and The Netherlands. Clin J Am Soc Nephrol. 2013; 8: 1685–1693. doi: 10.2215/CJN.12521212 23813554 10.2215/CJN.12521212 PMC3789356

[32] Crews DC, Charles RF, Evans MK, Zonderman AB, Powe NR. Poverty, race, and CKD in a racially and socioeconomically diverse urban population. Am J Kidney Dis. 2010; 55(6):992–1000. doi: 10.1053/j.ajkd.2009.12.032 20207457 10.1053/j.ajkd.2009.12.032 PMC2876201

[33] Martins D, Tareen N, Zadshir A, Pan D, Vargas R, Nissenson A, et al The association of poverty with the prevalence of albuminuria: data from the Third National Health and Nutrition Examination Survey (NHANES III). Am J Kidney Dis. 6 2006; 47(6): 965–971. doi: 10.1053/j.ajkd.2006.02.179 16731291 10.1053/j.ajkd.2006.02.179 PMC3863615

[34] McClellan WM, Newsome BB, McClure LA, Howard G, Volkova N, Audhya P, et al Poverty and racial disparities in kidney disease: the REGARDS study. Am J Nephrol. 2010; 32(1):38–46. doi: 10.1159/000313883 20516678 10.1159/000313883 PMC2914392

[35] Evans K, Coresh J, Bash LD, Gary-Webb T, Kottgen A, Carson K, et al Race differences in access to health care and disparities in incident chronic kidney disease in the US. Nephrol Dial Transplant. 2011; 26(3): 899–908. doi: 10.1093/ndt/gfq473 20688771 10.1093/ndt/gfq473 PMC3108345

[36] Hall YN, Rodriguez RA, Boyko EJ, Chertow GM, O’Hare AM. Characteristics of uninsured Americans with chronic kidney disease. J Gen Intern Med. 2009; 24(8): 917–922. doi: 10.1007/s11606-009-1028-3 19506974 10.1007/s11606-009-1028-3 PMC2710472

[37] Wakamatsu-Yamanaka T, Fukuda M, Sato R, Naito T, Togawa H, Tomonari T, et al Geographic differences in the increasing ESRD rate have disappeared in Japan. Clin Exper Nephrol. 2011; 15: 708–713. 21638004 10.1007/s10157-011-0466-5

[38] Crews DC, Liu Y, Boulware LE. Disparities in the burden, outcomes and care of chronic kidney disease. Curr Opin Nephrol Hypertens. 2014; 23(3): 298–305. doi: 10.1097/01.mnh.0000444822.25991.f6 24662984 10.1097/01.mnh.0000444822.25991.f6 PMC4126677

[39] Centers for Disease Control and Prevention (CDC). Vital signs: Decrease in incidence of diabetes-related end-stage renal disease among American Indians/Alaska Natives—United States, 1996–2013. MMWR Morb Mortal Wkyl Rep. 2017; 66: 1–7. 10.15585/mmwr.mm6601e1 PMC5687264 28081061

[40] Norris KC, Mensah GA, Boulware LE, Lu JL, Ma JZ, Streja E, et al Age, race and cardiovascular outcomes in African American veterans. Ethn Dis. 2016; 26(3): 305–14. doi: 10.18865/ed.26.3.305 27440969 10.18865/ed.26.3.305 PMC4948796

[41] Menon V, Gul, Sarnak MJ. Cardiovascular risk factors in chronic kidney disease. Kidney Int. 2005; 68: 1413–1418. doi: 10.1111/j.1523-1755.2005.00551.x 16164615 10.1111/j.1523-1755.2005.00551.x

[42] Levey AS, Stevens LA, Coresh J. Conceptual model of CKD: Applications and implications. Am J Kidney Dis. 2009; 53(3, Supp3): S4–S16. 19231760 10.1053/j.ajkd.2008.07.048

