# Supplementary material for: Evidence of chronic kidney disease in veterans with incident diabetes mellitus
Source: PLoS One. 2018 Feb 9;13(2):e0192712. doi: 10.1371/journal.pone.0192712 (PMC5806889; doi:10.1371/journal.pone.0192712)
Supplement: S1 Appendix — (DOCX) [file pone.0192712.s001.docx]

**S1 Appendix. Diagnostic and Procedural Codes**

Exclusion Codes*

HIV: 042-044.9, V08, 795.71

Malignant cancer: 140-172.9, 174-195.8, 200-208.9

Retinopathy: 250.5, 362.0x

Neuropathy: 250.6, 357.2

Foot ulcer: 707.1x

Nephropathy: 250.4x

End-stage renal disease: 585.6

Codes for Comorbidities*

Cerebrovascular Disease: 430-438

Myocardial Infarction: 410-410.9, 412
Peripheral Arterial Disease: 440.0-440.9, 443.x, 38.0, 38.1, 39.50, 39.22, 39.24, 39.25, 39.26, 29.28
Chronic obstructive Pulmonary Disease: 490-496, 500-505, 506.4

Congestive Heart Failure: 428-428.9

*All codes listed are ICD-9 CM
