# Supplementary figures and images for: Evidence of chronic kidney disease in veterans with incident diabetes mellitus
Source: PLoS One. 2018 Feb 9;13(2):e0192712. doi: 10.1371/journal.pone.0192712 (PMC5806889; doi:10.1371/journal.pone.0192712)

**S2 Appendix. Logistic Regression Sensitivity Analysis**


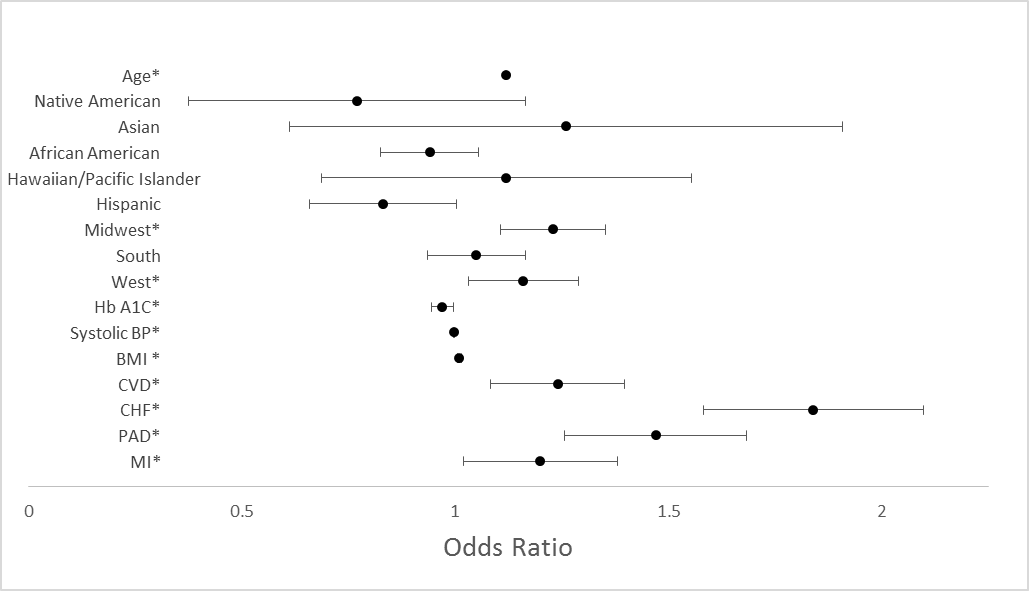


*p<0.05

Supplement: S2 Appendix — (DOCX) [file pone.0192712.s002.docx]
